# Surgical and postoperative management of ulnar neuropathy at the elbow: a national survey among Dutch hand and neurosurgeons

**DOI:** 10.1016/j.bas.2026.106662

**Published:** 2026-09-01

**Authors:** Isabelle M.L.P. Kamm, Nadine Boers, Godard C.W. de Ruiter, J. Henk Coert

**Affiliations:** a Department of Plastic, Reconstructive and Hand Surgery, University Medical Center Utrecht, Utrecht, the Netherlands; b Department of Neurosurgery, Haaglanden Medical Center, The Hague, the Netherlands

**Keywords:** Ulnar neuropathy, Ulnar nerve surgery, Open decompression, Transposition surgery, Postoperative management

## Abstract

**Introduction:**

Ulnar neuropathy at the elbow (UNE) is a condition for which multiple surgical techniques exist, yet no consensus has been reached on the optimal approach. Additionally, postoperative follow-up protocols are not standardized, resulting in considerable variability in clinical practice.

**Research question:**

To better understand current practices, a nationwide survey was conducted among Dutch surgeons involved in the surgical management of UNE.

**Materials and methods:**

An online questionnaire was sent to all plastic surgeons, neurosurgeons and orthopedic surgeons with expertise in hand surgery in the Netherlands. The survey assessed surgical preferences, postoperative follow-up routines, and rehabilitation practices for both primary and revision UNE cases.

**Results:**

In total, 163 surgeons filled out the survey. Open decompression was the preferred surgical technique for primary UNE (92.6%), subcutaneous transposition was most commonly selected for revision procedures (37.9%). The initial postoperative consultation was typically scheduled 4-8 weeks after surgery (55.2%). In case of persistent and recurrent UNE, repeated physical appointments were the most frequently chosen follow-up strategy (82.6% and 63.4%, respectively). For recurrent UNE, 31.7% opted for immediate diagnostic testing, with 64.7% selecting both electromyography and ultrasound as the preferred diagnostic approach. Postoperative splinting was rarely advised (0.6%). Hand therapy was recommended by 66.3% of surgeons and usually initiated within the first four weeks postoperatively (54.6%).

**Discussion and conclusion:**

While there is broad agreement on the use of open decompression for primary UNE, notable variation remains in revision procedures and follow-up strategies. These findings underscore the need for standardized, evidence-based protocols to support consistent and effective care for UNE patients.

## Highlights

• Open decompression is the preferred surgical treatment for cubital tunnel syndrome.
• Subcutaneous transposition is most frequently chosen in revision surgery.
• Follow-up practices after ulnar nerve surgery vary widely between surgeons.
• Absence of consensus on postoperative care emphasizes need for national guidelines.

## 1 Introduction

Ulnar neuropathy at the elbow (UNE) is the second most prevalent entrapment neuropathy in the upper extremity after carpal tunnel syndrome, with an annual incidence of 20.9 per 100,000 person years (Mondelli et al., 2005). According to the Dutch guideline concerning UNE, patients with mild symptoms should initially receive conservative treatment. Surgery is recommended if conservative treatment fails, or if symptoms are too severe to justify a non-surgical approach. (Kennisinstituut van de Federatie van Medisch Specialisten - Richtlijnen database) Several surgical techniques are available for the treatment of UNE, including simple decompression, anterior transposition of the ulnar nerve with placement either subcutaneously, submuscularly or intramuscularly, and medial epicondylectomy (Robertson and Saratsiotis, 2005). Although the national guideline provides general recommendations on UNE treatment indications, it offers limited guidance on the choice between different surgical techniques, based on low-certainty evidence. The choice of the procedure often depends on the degree of nerve compression, anatomical considerations, and surgeon preference and experience. Still no clear multidisciplinary consensus exists on the optimal surgical treatment for UNE (Assmus and Antoniadis, 2024; Caliandro et al., 2025).

Although surgical intervention for UNE is generally effective, revision surgery is required in up to 18% of cases (Smit et al., 2023). Given this risk of recalcitrant UNE, appropriate postoperative follow-up is essential for monitoring recovery and identifying patients who may require further intervention. Although consensus-based recommendations on follow-up after UNE surgery have recently been proposed by the Core Outcomes in Nerve Surgery (COINS) consortium, these recommendations are focused on standardized outcome measures for research purposes and do not, for example, provide guidance on postoperative electrophysiologic or ultrasound (US) studies in case of persistence of symptoms (Wilson et al., 2024). After surgical treatment of carpal tunnel syndrome, neuroanatomical normalization has been observed between four weeks and three months postoperatively (Li et al., 2019). However, in the absence of a standardized, multidisciplinary postoperative management and follow-up protocol for UNE, potential recovery patterns of the ulnar nerve remain unexplored. Currently, the lack of a structured follow-up protocol leaves physicians free to determine their own approaches, resulting in considerable variation in terms of frequency and timing of follow-up care. In addition to variability in postoperative management, differences may also exist in the choice of surgical technique, both for primary and revision surgeries (Natroshvili et al., 2019; Wever et al., 2018). To better understand the current clinical practice in the Netherlands, a questionnaire study was conducted. The aim of the study was to assess the surgical treatment and follow-up patterns of patients following cubital tunnel surgery and to determine the extent of their variation among Dutch plastic surgeons, neurosurgeons and orthopedic surgeons.

## 2 Methods

### 2.1 Questionnaire

An online questionnaire was developed using REDCap (Research Electronic Data Capture), a secure, web-based platform for building and managing surveys and databases. The aim of the questionnaire was to assess current clinical practices related to the surgical management and postoperative care of patients with UNE. The survey was administered in Dutch (for the version translated to English: see Appendix) and was structured into three main sections: (1) preferred surgical techniques for both primary and persistent or recurrent UNE, (2) postoperative follow-up strategies, including the timing and type of follow-up visits, and the management of persistent and recurrent symptoms, and (3) rehabilitation practices, including the use of splinting and hand therapy.

The survey was distributed via email to all plastic surgeons, neurosurgeons and orthopedic surgeons with a hand surgery sub-specialization in the Netherlands. Distribution was facilitated through the Dutch Society of Plastic Surgery (NVPC), the Dutch Society for Neurosurgery (NVvN), and the Shoulder & Elbow and Hand & Wrist working groups of the Dutch Orthopaedic Association (NOV). To support participation, one follow-up email was sent during the data-collection period. Survey responses were collected between January 24, 2025 and April 4, 2025. Data collection took place anonymously as no personal identifying information was required.

### 2.2 Data analysis

The evaluation of the questionnaire results was primarily of descriptive nature. Data were summarized using frequencies and percentages to characterize the distribution of surgical preferences and postoperative practices. Statistical analyses and data visualizations were performed using R Statistical Software, Version 4.4.2 (R Core Team, 2024).

## 3 Results

A total of 163 respondents completed the survey. Among these, 85 (52.1%) were plastic surgeons, 57 (35.0%) neurosurgeons and 21 (12.9%) orthopedic surgeons. Details on type of hospital and practice are shown in Table 1.

**Table 1:** Hospital and practice type of respondents.

Hospital or practice type	Plastic surgeons (n = 85)	Neurosurgeons (n = 57)	Orthopedic surgeons (n = 21)	All surgeons (N = 163)
Academic hospital	15.3% (13/85)	47.7% (27/57)	14.3% (3/21)	26.4% (43/163)
Peripheral, teaching hospital	38.8% (33/85)	28.1% (16/57)	38.1% (8/21)	35.0% (57/163)
Peripheral, non-teaching hospital	31.8% (27/85)	19.3% (11/57)	23.8% (5/21)	26.4% (43/163)
Independent treatment center or private clinic	11.8% (10/85)	1.8% (1/57)	14.3% (3/21)	8.6% (14/163)
Both academic and peripheral hospital	1.2% (1/85)	3.5% (2/57)	-	1.8% (3/163)
Both teaching hospital and independent treatment center	-	-	4.8% (1/21)	0.6% (1/163)
Other, not further specified	1.2% (1/85)	-	-	0.6% (1/163)
Missing	-	-	4.8% (1/21)	0.6% (1/163)

### 3.1 Surgical treatment

Overall, open decompression of the ulnar nerve was the most frequently chosen technique in case of primary UNE (92.6%). In case of persistent or recurrent symptoms following previous surgery, 37.9% of surgeons opted for subcutaneous transposition, 19.9% for intramuscular transposition, and 18.6% selected open decompression (Table 2).

**Table 2:** Surgical treatment and follow-up practices after UNE surgery.

	Plastic surgeons (n = 85)	Neurosurgeons (n = 57)	Orthopedic surgeons (n = 21)	All surgeons (N = 163)
**First choice surgical treatment – primary UNE**				
Decompression, open	88.2% (75/85)	98.2% (56/57)	95.2% (20/21)	92.6% (151/163)
Decompression, endoscopic	9.4% (8/85)	-	-	4.9% (8/163)
Subcutaneous transposition	-	1.8% (1/57)	-	0.6% (1/163)
Submuscular transposition, by Learmonth	-	-	4.8% (1/21)	0.6% (1/163)
Submuscular transposition, by Dellon	-	-	-	-
Intramuscular transposition	2.4% (2/85)	-	-	1.2% (2/163)
Medial epicondylectomy	-	-	-	-
**First choice surgical treatment – persistent/recurrent UNE**				
(Revision) decompression, open	18.1% (15/83)	22.8% (13/57)	9.5% (2/21)	18.6% (30/161)
(Revision) decompression, endoscopic	-	-	-	-
Subcutaneous transposition	36.1% (30/83)	36.8% (21/57)	47.6% (10/21)	37.9% (61/161)
Submuscular transposition, by Learmonth	7.2% (6/83)	14.0% (8/57)	19.0% (4/21)	11.2% (18/161)
Submuscular transposition, by Dellon	15.7% (13/83)	10.5% (6/57)	4.8% (1/21)	12.4% (20/161)
Intramuscular transposition	22.9% (19/83)	15.8% (9/57)	19.0% (4/21)	19.9% (32/161)
**Follow-up protocol at institution**				
No	41.2% (35/85)	42.1% (24/57)	47.6% (10/21)	42.3% (69/163)
Yes	50.8% (50/85)	57.9% (33/57)	52.4% (11/21)	57.7% (94/163)
***Adherence to this protocol***				
No	2.0% (1/49)	-	9.1% (1/11)	2.2% (2/92)
Yes	98.0% (48/49)	100% (32/32)	90.9% (10/11)	97.8% (90/92)
**Timing of first postoperative appointment**				
0-4 weeks post-surgery	58.8% (50/85)	5.3% (3/57)	33.3% (7/21)	36.8% (60/163)
4-8 weeks post-surgery	28.2% (24/85)	93.0% (53/57)	61.9% (13/21)	55.2% (90/163)
3 months post-surgery	11.8% (10/85)	-	4.8% (1/21)	6.7% (11/163)
6 months post-surgery	-	-	-	-
Other	1.2% (1/85)	1.8% (1/57)	-	1.2% (2/163)
**Type of first postoperative appointment**				
Physical appointment	96.5% (82/85)	42.1% (24/57)	90.5% (19/21)	76.7% (125/163)
Telephone consultation	3.5% (3/85)	57.9% (33/57)	9.5% (2/21)	23.3% (38/163)
**Management of persistent UNE**				
Frequent physical appointments	88.2% (75/85)	72.7% (40/55)	85.7% (18/21)	82.6% (133/161)
***Time interval between first and second appointment***				
0-4 weeks	5.3% (4/75)	-	5.6% (1/18)	3.8% (5/133)
4-8 weeks	24.0% (18/75)	27.5% (11/40)	33.3% (6/18)	26.3% (35/133)
3 months	54.7% (41/75)	65.0% (26/40)	55.6% (10/18)	57.9% (77/133)
6 months	14.7% (11/75)	7.5% (3/40)	5.6% (1/18)	11.3% (15/133)
Other	1.3% (1/75)	-	-	0.8% (1/133)
Frequent telephone consultations	8.2% (7/85)	21.8% (12/55)	14.3% (3/21)	13.7% (22/161)
***Time interval between first and second appointment***				
0-4 weeks	-	-	-	-
4-8 weeks	14.3% (1/7)	16.7% (2/12)	-	13.6% (3/22)
3 months	71.4% (5/7)	75.0% (9/12)	100% (3/3)	77.3% (17/22)
6 months	14.3% (1/7)	8.3% (1/12)	-	9.1% (2/22)
Other	-	-	-	-
Diagnostic testing	3.5% (3/85)	5.5% (3/55)	-	3.7% (6/161)
***First choice of diagnostic test***				
Electromyography	33.3% (1/3)	66.7% (2/3)	NA	50.0% (3/6)
Ultrasound	-	-	NA	-
Both electromyography and ultrasound	66.7% (2/3)	33.3% (1/3)	NA	50.0% (3/6)
Other	-	-	NA	-
**Management of recurrent UNE**				
Frequent physical appointments	60.7% (51/84)	69.6% (39/56)	57.1% (12/21)	63.4% (102/161)
Frequent telephone consultations	2.4% (2/84)	8.9% (5/56)	4.8% (1/21)	5.0% (8/161)
Diagnostic testing	36.9% (31/84)	21.4% (12/56)	38.1% (8/21)	31.7% (51/161)
***First choice of diagnostic test***				
Electromyography	12.9% (4/31)	16.7% (2/12)	25.0% (2/8)	15.7% (8/51)
Ultrasound	19.4% (6/31)	-	25.0% (2/8)	15.7% (8/51)
Both electromyography and ultrasound	61.3% (19/31)	83.3% (10/12)	50.0% (4/8)	64.7% (33/51)
Other	6.5% (2/31)	-	-	3.9% (2/51)
**Total follow-up duration**				
Only follow-up in case of symptoms	44.0% (37/84)	71.4% (40/56)	52.4% (11/21)	54.7% (88/161)
Up to 6 months post-surgery	38.1% (32/84)	17.9% (10/56)	38.1% (8/21)	31.1% (50/161)
Up to 1 year post-surgery	17.9% (15/84)	10.7% (6/56)	9.5% (2/21)	14.3% (23/161)
Up to 2 years post-surgery	-	-	-	-
Up to 3 years post-surgery	-	-	-	-
Up to 4 years post-surgery	-	-	-	-
Up to 5 years post-surgery	-	-	-	-

Abbreviations: UNE, ulnar neuropathy at the elbow.

**Fig. 1:**
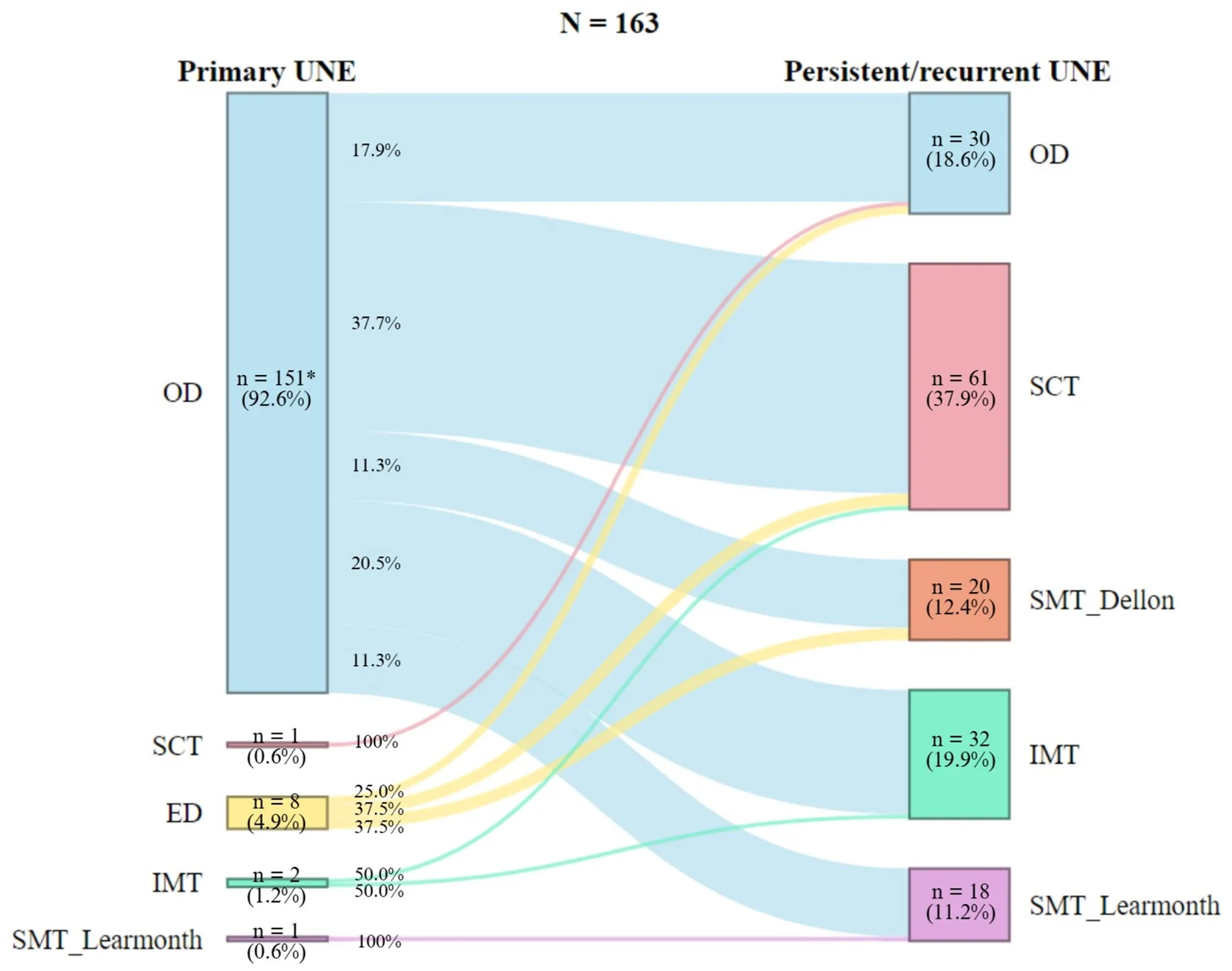
Sankey diagram of the surgical treatment choices for UNE. * Data on revision surgery preference after primary open decompression are missing for 2 of 151 respondents (1.3%) Abbreviations: ED, endoscopic decompression; IMT, intramuscular transposition; OD, open decompression; SCT, subcutaneous transposition; SMT, submuscular transposition.

The distribution of treatment preferences from primary to revision surgeries is visualized in Fig. 1. Among surgeons who initially chose open decompression, subcutaneous transposition was the most frequently preferred revision technique (37.7%).

### 3.2 Follow-up practice

More than half of the respondents indicated having a standardized postoperative follow-up protocol at their institution (57.7%). The first follow-up appointment typically occurred between four and eight weeks postoperatively (55.2%) and was most often conducted in person (76.7%). For patients experiencing persistent symptoms of UNE following surgery, 82.6% of respondents reported scheduling frequent physical appointments. The most common time interval between first and second consultation was three months (57.9%). In case of recurrent symptoms of UNE, most surgeons also relied on physical follow-up (63.4%). Nearly one-third opted for immediate diagnostic testing (31.7%), with both EMG and US being the most frequently chosen investigations (64.7%). In 54.7% of cases, surgeons indicated that additional follow-up after the initial postoperative assessment was only performed in patients reporting symptoms (Table 2).

### 3.3 Splinting and hand therapy

Routine postoperative splinting was rarely recommended (0.6%). In contrast, views on postoperative hand therapy varied. While 20.9% of surgeons routinely advised hand therapy, 33.7% did not, and 45.4% recommended it only when indicated. The most frequently reported indications included persistent symptoms, functional impairment, pre- or postoperative motor dysfunction, and movement anxiety. Hand therapy was generally initiated within the first four weeks after surgery (54.6%) and was advised most frequently following transposition procedures (85.3%) (Table 3).

**Table 3:** Splinting and hand therapy after UNE surgery.

	Plastic surgeons (n = 85)	Neurosurgeons (n = 57)	Orthopedic surgeons (n = 21)	All surgeons (N = 163)
**Splint application post-surgery**				
No	98.8% (84/85)	100% (57/57)	100% (21/21)	99.4% (162/163)
Yes	1.2% (1/85)	-	-	0.6% (1/163)
**Referral to hand therapist post-surgery**				
No	28.2% (24/85)	38.6% (22/57)	42.9% (9/21)	33.7% (55/163)
Only when indicated	36.5% (31/85)	59.6% (34/57)	42.9% (9/21)	45.4% (74/163)
Yes	35.3% (30/85)	1.8% (1/57)	14.3% (3/21)	20.9% (34/163)
***Type of surgery after which hand therapy is advised***^a^				
Decompression	66.7% (20/30)	100% (1/1)	66.7% (2/3)	67.6% (23/34)
Transposition	86.7% (26/30)	100% (1/1)	66.7% (2/3)	85.3% (29/34)
Medial epicondylectomy	13.3% (4/30)	-	-	11.8% (4/34)
Revision surgery	60.0% (18/30)	100% (1/1)	66.7% (2/3)	61.8% (21/34)
**Timing of hand therapy**				
0-4 weeks post-surgery	70.5% (43/61)	17.1% (6/35)	83.3% (10/12)	54.6% (59/108)
4-8 weeks post-surgery	21.3% (13/61)	51.4% (18/35)	16.7% (2/12)	30.6% (33/108)
3 months post-surgery	1.6% (1/61)	22.9% (8/35)	-	8.3% (9/108)
6 months post-surgery	1.6% (1/61)	2.9% (1/35)	-	1.9% (2/108)
Other	4.9% (3/61)	5.7% (2/35)	-	4.6% (5/108)

a This was a checkbox question which allowed respondents to select multiple answers.

## 4 Discussion

This nationwide survey shows a comprehensive overview of the current surgical treatment and follow-up practices for patients with UNE among Dutch plastic surgeons, neurosurgeons and orthopedic surgeons. The results show considerable variation in surgical approaches and follow-up strategies. Although guidelines for UNE exist in the Netherlands and other countries, including Germany, they are largely based on available literature of low-to moderate-quality evidence and, as a result, offer no specific guidance regarding electrophysiological or sonographic follow-up, or the choice of surgical technique in cases of persistent or recurrent symptoms. (Kennisinstituut van de Federatie van Medisch Specialisten - Richtlijnen database; Arbeitsgemeinschaft der Wissenschaftlichen Medizinischen Fachgesellschaften (AWMF), 2018) Consequently, these guidelines may provide insufficient direction to standardize clinical practice, allowing variation in treatment strategies to persist.

The majority of surgeons in our study reported using open in situ decompression of the ulnar nerve as their preferred surgical treatment for primary UNE (151/163, 92.6%). This finding is in line with previous research in which open decompression was identified as the most selected technique among 1069 surgeons in the United States (Yahya et al., 2018). The widespread adoption of open decompression of the ulnar nerve in both our cohort and previous studies can be attributed to several advantages. Open decompression is a relatively simple procedure involving limited surgical dissection, which reduces the risk of ulnar nerve devascularization (Ogata et al., 1984). Moreover, it is associated with shorter operation times, lower complication rates (Bartels et al., 2005a; Biggs and Curtis, 2006), lower costs (Bartels et al., 2005b), and faster recovery (Bartels et al., 2005a). Clinical outcomes, including symptom improvement, patient satisfaction and electrophysiological improvement, have been shown to be comparable to those of more invasive techniques such as anterior transposition (Zlowodzki et al., 2007; Keiner et al., 2009). Anterior transposition may be favored in patients exhibiting ulnar nerve (sub)luxation (Bartels et al., 1998). However, it has not been shown conclusively that the presence of (sub)luxation should dictate the choice of surgical technique, with some evidence suggesting comparable outcomes between open decompression and subcutaneous transposition even when (sub)luxation is present (Bartels et al., 2005a). Hutchinson et al., 2021 found significantly higher revision rates for in situ decompression compared to subcutaneous transposition, particularly in younger patients and women (Keiner et al., 2009). Their findings underscore the importance of a patient-specific approach, balancing the general advantages of open decompression against the potential risk of recurrence in select populations.

Despite the general consensus regarding primary surgery, our study revealed a more heterogeneous approach to revision procedures. As the optimal management of failed primary procedures remains controversial, the choice of revision technique often depends on the experience and preference of the surgeon. When looking specifically at patterns of primary and revision procedures, our data show that most surgeons perform subcutaneous transposition after failed primary open decompression (57/151, 37.7%). This strategy is in line with the surgical algorithm proposed by Grandizio et al. (2018), where subcutaneous transposition is recommended as the revision technique following failed primary in situ decompression (Bartels et al., 1998). Our findings provide insight into prevailing surgical preferences but do not allow conclusions regarding the relative efficacy of different revision strategies. Techniques such as submuscular transposition, although reported less frequently, may be reserved for more complex or recurrent cases, and their role in surgical decision-making should not be understated. The choice between revision techniques is likely influenced by a combination of anatomical and procedural factors, as well as differences in recovery time and the potential for scar tissue formation (De Ruiter et al., 2020). These considerations may affect surgical preference, yet comparative evidence on outcomes between techniques is currently lacking. It also remains unclear to what extent these different options, along with their respective advantages and disadvantages, are routinely discussed with patients during preoperative counselling.

Regarding postoperative follow-up practices, most respondents have a standardized institutional protocol (94/163, 57.7%). However, there is a clear variation between practices and surgeons, suggesting that surgeons may adapt their protocolized follow-up schedules based on clinical judgment or patient-specific factors. In cases of recurrent symptoms, the majority of respondents reported relying primarily on repeated physical examinations (102/161, 63.4%), while 31.7% opted for diagnostic testing. Within this group, 64.7% would request both electromyography (EMG) and US to assist in decision-making. However, the value of these findings in the context of recurrent or persistent symptoms remains a matter of debate. Whereas electrodiagnostic studies and US in primary UNE yield sensitivities of 63.3% (Van Veen et al., 2015) and 85% (Chang et al., 2018), respectively, Boers et al. (2022) reported lower values of 40.7% and 71.4% in patients with persistent or recurrent symptoms following prior ulnar nerve surgery. Although US may provide insight into postoperative anatomy and appearance of the nerve, both EMG and US should be critically appraised. As emphasized in that same study (Boers et al., 2022), the diagnosis of recurrent or persistent UNE should primarily be based on clinical assessment, with diagnostic testing serving a supportive rather than decisive role. To date, no studies have investigated the prognostic value of EMG and US in guiding revision surgery for recurrent or persistent UNE.

The relatively frequent use of US observed in our cohort likely reflects national practice patterns in the Netherlands, where US is widely accessible and routinely used in the evaluation of UNE. International expert consensus similarly supports US alongside electrodiagnostic studies in the diagnostic assessment of UNE (Pelosi et al., 2021). In contrast, magnetic resonance imaging (MRI) is considered the reference standard for imaging UNE by the American College of Radiology (Thomas et al., 2022), and may be more frequently incorporated into the diagnostic work-up in other countries. A recent cost-effectiveness study suggests that initial US followed by MRI is the most cost-effective approach (Jardon et al., 2025). However, in the absence of robust comparative evidence, it remains unclear which imaging modality is superior. Consequently, imaging choices in clinical practice are largely driven by pragmatic factors, such as availability, expertise and institutional protocols, and may differ across countries.

Postoperative rehabilitation strategies also showed considerable variability. Most respondents did not recommend routine postoperative splinting (162/163, 99.4%). This is in line with current literature suggesting that prolonged immobilization does not reduce pain or adverse events, and may even delay functional recovery and returning to work, with an average increase of 40 days compared to early mobilization (Olaiya et al., 2025). In line with these findings, most surgeons recommended hand therapy (108/163, 66.3%), particularly after transposition surgeries. Early initiation of hand therapy within four weeks postoperatively was most frequently reported. Given that early mobilization has been associated with faster recovery and reduced time of work, structured rehabilitation protocols should be considered to enhance postoperative recovery and optimize functional outcomes.

A limitation of our study lies in the inability to calculate the exact response rate, due to the unknown total number of surgeons in the Netherlands performing ulnar nerve surgery. However, since we were able to collect data from a large group of surgeons from different types of hospitals (i.e. academic, peripheral teaching, peripheral non-teaching and private practices), we expect that we included a heterogenous group of surgeons, representative of all surgeons in the Netherlands.

## 5 Conclusion

This nationwide survey highlights variation in surgical and postoperative management of UNE among Dutch surgeons. Open decompression of the ulnar nerve is the preferred treatment for primary UNE, reflecting evidence from clinical trials and international practice. However, revision strategies are more diverse, with 37.9% of surgeons opting for subcutaneous transposition, 23.6% for submuscular transposition using different techniques (i.e. Dellon or Learmonth), 19.9% for intramuscular transposition, and 18.6% for open decompression. Follow-up practices also show considerable heterogeneity, despite the availability of institutional protocols. These findings underscore the need for multidisciplinary consensus guidelines to support more uniform, evidence-based decision-making in both surgical treatment and postoperative care of UNE.

## Informed consent

Not applicable.

## Ethical approval

Not applicable.

## Contributorship

Isabelle M.L.P. Kamm: investigation, methodology, project administration, formal analysis, visualization, writing – original draft preparation, writing – review and editing.

Nadine Boers: conceptualization, supervision, writing – review and editing.

Godard C.W. de Ruiter: conceptualization, supervision, writing – review and editing.

J. Henk Coert: conceptualization, supervision, writing – review and editing.

## Funding

This research did not receive any specific grant from funding agencies in the public, commercial, or not-for-profit sectors.

## Declaration of competing interests

The authors declare that they have no known competing financial interests or personal relationships that could have appeared to influence the work reported in this paper.

## References

[bib1] Arbeitsgemeinschaft der Wissenschaftlichen Medizinischen Fachgesellschaften (AWMF) (2018). Diagnostik und Therapie des Kubitaltunnelsyndroms (KUTS). Leitlinienreport. https://register.awmf.org/assets/guidelines/005-009m_S3_Kubitaltunnelsyndrom-Diagnostik-Therapie_2018-02-abgelaufen.pdf.

[bib2] Assmus H., Antoniadis G. (2024). Nerve Compression Syndromes: a Practical Guide.

[bib3] Bartels R.H.M.A., Menovsky T., Van Overbeeke J.J., Verhagen W.I.M. (1998). Surgical management of ulnar nerve compression at the elbow: an analysis of the literature. J. Neurosurg..

[bib5] Bartels R.H.M.A., Termeer E.H., Van Der Wilt G.J., Van Rossum L.G.M., Meulstee J., Verhagen W.I.M. (2005). Simple decompression or anterior subcutaneous transposition for ulnar neuropathy at the elbow: a cost-minimization analysis - part 2. Neurosurgery.

[bib4] Bartels R.H.M.A., Verhagen W.I.M., Van Der Wilt G.J., Meulstee J., Van Rossum L.G.M., Grotenhuis J.A. (2005). Prospective randomized controlled study comparing simple decompression versus anterior subcutaneous transposition for idiopathic neuropathy of the ulnar nerve at the elbow: part 1. Neurosurgery.

[bib6] Biggs M., Curtis J.A. (2006). Randomized, prospective study comparing ulnar neurolysis in situ with submuscular transposition. Neurosurgery.

[bib7] Boers N., Brakkee E.M., Krijgh D.D., Coert J.H. (2022). The diagnostic role of ultrasound in cubital tunnel syndrome for patients with a previous cubital tunnel surgery. J. Plast. Reconstr. Aesthetic Surg..

[bib8] Caliandro P., La Torre G., Padua R., Giannini F., Reale G., Padua L. (2025). Treatment for ulnar neuropathy at the elbow. Cochrane Database Syst. Rev..

[bib9] Chang K.-V., Wu W.-T., Han D.-S., Özçakar L. (2018). Ulnar nerve cross-sectional area for the diagnosis of cubital tunnel syndrome: a meta-analysis of ultrasonographic measurements. Arch. Phys. Med. Rehabil..

[bib10] De Ruiter G.C.W., Verhamme C., Coert J.H., Coert B.A. (2020). Diagnose en behandeling van ulnaropathie bij de elleboog. Tijdschr Neurol Neurochir.

[bib11] Grandizio L.C., Maschke S., Evans P.J. (2018). The management of persistent and recurrent cubital tunnel syndrome. J. Hand Surg..

[bib12] Hutchinson D.T., Sullivan R., Sinclair M.K. (2021). Long-term reoperation rate for cubital tunnel syndrome: subcutaneous transposition versus in situ decompression. Hand.

[bib13] Jardon M., Subhas N., Sneag D.B., Li Z.I., Jazrawi L.M., Paksima N. (2025). Diagnostic workup of ulnar neuropathy at the elbow: a cost-effectiveness study. Acad. Radiol..

[bib14] Keiner D., Gaab M.R., Schroeder H.W.S., Oertel J. (2009). Comparison of the long-term results of anterior transposition of the ulnar nerve or simple decompression in the treatment of cubital tunnel syndrome-a prospective study. Acta Neurochir..

[bib15] Kennisinstituut van de Federatie van Medisch Specialisten - Richtlijnen database. Nederlandse Richtlijn Ulnaropathie. https://richtlijnendatabase.nl/richtlijn/ulnaropathie/ulnaropathie_-_startpagina.html. .

[bib16] Li M., Jiang J., Zhou Q., Zhang C. (2019). Sonographic follow-up after endoscopic carpal tunnel release for severe carpal tunnel syndrome: a one-year neuroanatomical prospective observational study. BMC Muscoskelet. Disord..

[bib17] Mondelli M., Giannini F., Ballerini M., Ginanneschi F., Martorelli E. (2005). Incidence of ulnar neuropathy at the elbow in the province of Siena (Italy). J. Neurol. Sci..

[bib18] Natroshvili T., Walbeehm E.T., Van Alfen N., Bartels R.H.M.A. (2019). Results of reoperation for failed ulnar nerve surgery at the elbow: a systematic review and meta-analysis. J. Neurosurg..

[bib19] Ogata K., Manske P.R., Lesker P.A. (1984). The effect of surgical dissection on regional blood flow to the ulnar nerve in the cubital tunnel. Clin. Orthop. Relat. Res..

[bib20] Olaiya O.R., Huynh M., Ebeye T., Gallo L., Mbuagbaw L., McRae M. (2025). Immediate versus delayed mobilization after cubital tunnel release surgery: a systematic review and meta-analysis. Plastic Surgery.

[bib21] Pelosi L., Arányi Z., Beekman R., Bland J., Coraci D., Hobson-Webb L.D. (2021). Expert consensus on the combined investigation of ulnar neuropathy at the elbow using electrodiagnostic tests and nerve ultrasound. Clin. Neurophysiol..

[bib22] Robertson C., Saratsiotis J. (2005). A review of compressive ulnar neuropathy at the elbow. J. Manip. Physiol. Ther..

[bib23] Smit J.A., Hu Y., Brohet R.M., Van Rijssen A.L. (2023). Identifying risk factors for recurrence after cubital tunnel release. J. Hand Surg..

[bib24] Thomas J.M., Chang E.Y., Ha A.S., Bartolotta R.J., Bucknor M.D., Caracciolo J.T. (2022). ACR appropriateness criteria® chronic elbow pain. J. Am. Coll. Radiol..

[bib25] Van Veen K.E.B., Wesstein M., Van Kasteel V. (2015). Ultrasonography and electrodiagnostic studies in ulnar neuropathy: an examination of the sensitivity and specificity and the correlations between both diagnostic tools. J. Clin. Neurophysiol..

[bib26] Wever N., De Ruiter G.C.W., Coert J.H. (2018). Submuscular transposition with musculofascial lengthening for persistent or recurrent cubital tunnel syndrome in 34 patients. J Hand Surg Eur Vol.

[bib27] Wilson T.J., Davis G.A., Dengler N.F., Guedes F., Hébert-Blouin M.N., Jack M.M. (2024). Core outcomes in nerve surgery: development of a core outcome set for ulnar neuropathy at the elbow. J. Neurosurg..

[bib28] Yahya A., Malarkey A.R., Eschbaugh R.L., Bamberger H.B. (2018). Trends in the surgical treatment for cubital tunnel syndrome: a survey of members of the american society for surgery of the hand. Hand.

[bib29] Zlowodzki M., Chan S., Bhandari M., Kalliainen L., Schubert W. (2007). Anterior transposition compared with simple decompression for treatment of cubital tunnel syndrome: a meta-analysis of randomized, controlled trials. J. Bone Joint Surg..

