# Supplementary material for: Surgical and postoperative management of ulnar neuropathy at the elbow: a national survey among Dutch hand and neurosurgeons
Source: Brain Spine. 2026 Sep 1;6:106662. doi: 10.1016/j.bas.2026.106662 (PMC13572306; doi:10.1016/j.bas.2026.106662)
Supplement: Supplementary file 1 [file mmc1.docx]

**Appendix**

**Questionnaire**

**General**

1. What is your medical specialty?

- Neurosurgery
- Plastic surgery
- Orthopedic surgery
- Other [please specify]

2. What type of hospital are you working?

- Academic hospital
- Peripheral teaching hospital
- Peripheral non-teaching hospital
- Other [please specify]

3. Are you involved in the surgical treatment and follow-up care of patients with cubital tunnel syndrome?

- Yes
- No *🡪 End of survey*

**Treatment**

4. What is your first choice of surgical treatment for a patient with ulnar neuropathy without prior surgery, trauma, dislocation or anatomical variation on preoperative assessment?

- Ulnar nerve decompression, open
- Ulnar nerve decompression, endoscopic
- Ulnar nerve transposition – subcutaneously
- Ulnar nerve transposition – submuscularly, described by Learmonth
- Ulnar nerve transposition – submuscularly, described by Dellon
- Ulnar nerve transposition – intramuscularly
- Medial epicondylectomy

5. What is your first choice of surgical treatment for persistent or recurrent ulnar neuropathy following prior surgery performed by yourself?

- (Revision) ulnar nerve decompression, open
- (Revision) ulnar nerve decompression, endoscopic
- Ulnar nerve transposition – subcutaneously
- Ulnar nerve transposition – submuscularly, described by Learmonth
- Ulnar nerve transposition – submuscularly, described by Dellon
- Ulnar nerve transposition – intramuscularly

**Follow-up**

6. Does your institution have a standardized follow-up protocol for patients who have undergone surgery for cubital tunnel syndrome?

- Yes
  - 6.1. Do you adhere to this protocol?
    - Yes
    - No
      - 6.1.1. Why not?
        - [please specify]
- No

7. When is the first postoperative follow-up appointment scheduled (either physical appointment or telephone consultation)? *(This refers to an appointment with the surgeon, not an appointment for only suture removal.)*

- 0-4 weeks post-surgery
- 4-8 weeks post-surgery
- 3 months post-surgery
- 6 months post-surgery
- Other [please specify]

8. Is this usually a physical appointment or a telephone consultation?

- Physical appointment
- Telephone consultation

9. How do you manage patients with persistent symptoms of cubital tunnel syndrome after ulnar nerve surgery?

- Frequent physical appointments
  - 9.1. What is usually the time interval between the first and second appointment?
    - 0-4 weeks
    - 4-8 weeks
    - 3 months
    - 6 months
    - Other [please specify]
- Frequent telephone consultations
  - 9.1. What is usually the time interval between the first and second appointment?
    - 0-4 weeks
    - 4-8 weeks
    - 3 months
    - 6 months
    - Other [please specify]
- Diagnostic testing
  - 9.1.Which diagnostic tests do you usually request first?
    - Electromyography
    - Ultrasound
    - Both electromyography and ultrasound
    - Other [please specify]

10. How do you manage patients with recurrent symptoms of cubital tunnel syndrome after ulnar nerve surgery?

- Frequent physical appointments
- Frequent telephone consultations
- Diagnostic testing
  - 10.1. Which diagnostic tests do you usually request first?
    - Electromyography
    - Ultrasound
    - Both electromyography and ultrasound
    - Other [please specify]

11. What is the usual total follow-up duration after cubital tunnel syndrome surgery?

- Only follow-up in case of symptoms
- Up to 6 months post-surgery
- Up to 1 year post-surgery
- Up to 2 years post-surgery
- Up to 3 years post-surgery
- Up to 4 years post-surgery
- Up to 5 years post-surgery

**Splinting and hand therapy**

12. Are patients usually given a splint after ulnar nerve surgery?

- Yes
- No

13. Do you usually refer patients to a hand therapist after ulnar nerve surgery?

- Yes
  - 13.1. From what time post-surgery do you recommend starting hand therapy?
    - 0-4 weeks post-surgery
    - 4-8 weeks post-surgery
    - 3 months post-surgery
    - 6 months post-surgery
    - Other [please specify]
  - 13.2. After which type of ulnar nerve surgery do you typically recommend hand therapy? *(You may select multiple answers)*
    - Ulnar nerve decompression
    - Ulnar nerve transposition
    - Medial epicondylectomy
    - Revision surgery
- No
- Only when indicated
  - 13.1. When is hand therapy indicated?
    - [please specify]
  - 13.2. From what time post-surgery do you recommend starting hand therapy?
    - 0-4 weeks post-surgery
    - 4-8 weeks post-surgery
    - 3 months post-surgery
    - 6 months post-surgery
    - Other [please specify]
